# Supplementary material for: DNA binding drives the association of BRG1/hBRM bromodomains with nucleosomes
Source: Nat Commun. 2017 Jul 14;8:16080. doi: 10.1038/ncomms16080 (PMC5519978; doi:10.1038/ncomms16080)
Supplement: Supplementary Information [file ncomms16080-s1.pdf]

Title of file for HTML: Supplementary Information
Description: Supplementary Figures and Supplementary Tables

Title of file for HTML: Peer Review File
Description:

Peptide substrate	hBRM-A K_d (mM)		hBRM-B K_d (mM)		BRG1 K_d (mM)
	AT-BRD	BRD	AT-BRD	BRD	AT-BRD
**H3(9-19)K14ac**<br>KSTGG - Kac - APRKQ	0.81 ± 0.09	0.88 ± 0.09	0.76 ± 0.09<br>1.12 ± 0.14 *	0.90 ± 0.06	0.94 ± 0.12
**H3(18-28)K23ac**<br>KQLAT - Kac - AARKS				3.5 ± 1.4	
**H3(23-34)K27ac**<br>KAAR - Kac - SAPATGG	>35			>7	> 3
**H3(1-30)K(14,18,23,27)ac-GGK(biotin)**<br>ARTKQTARKSTGG - Kac - APR - Kac - QLAT - Kac - AAR - Kac - SAPGG - K(biotin)				0.35 ± 0.02	
**H4(1-20)K8ac**<br>SGRGKGG - Kac - GLGKGGAKRHRK	4 ± 5	3 ± 4	3 ± 2	2.8 ± 1.6	> 3
**H4(15-25)K20ac**<br>AKRHR - Kac - VLRDN				2.4 ± 0.9	
**Kac**	>50			>30	
**H3(5-23)**<br>QTARKSTGGKAPRKQLASK				>7	
**H3(23-34)**<br>KAARKSAPATGG				>16	

* $K_{d,\ obs}$ when pre-bound to DNAI

Supplementary Table 1. Dissociation constants (K_d) of hBRM/BRG1 BRD and AT-BRD for histone peptides, determined via NMR at 20°C (25°C for BRG1), 50mM potassium phosphate pH 7.0, 50mM KCl, 0.5mM EDTA, 1mM DTT. Shown are the averages over individual residues with significant CSPs (12-19 in total) and associated standard deviation.

DNA substrate		hBRM-A K_d (μM)	hBRM-B K_d (μM)			BRG1 K_d (μM)
		**BRD**	**AT-BRD**	**K1450D,K1451N, K1460A AT-BRD**	**BRD**	**AT-BRD**
**DNAI**	`CTCAATTGGT`<br>`GAGTTAACCA`	230 ± 60 †		100 ± 20	600 ± 200 †	
**DNAI.FA**	`*CCTCAATTGGTC`<br>`  GGAGTTAACCAG`		1.6 ± 0.4 ‡			16 ± 2 ‡
**ssDNAI.FA**	`*CCTCAATTGGTC`		42 ± 5 ‡			
**DNAII**	`CGTAGACAGCT`<br>`GCATCTGTCGA`	270 ± 60 †		100 ± 30	400 ± 100 †	
**DNAII.FA**	`*CGTAGACAGCTC`<br>`  GCATCTGTCGAG`		3.9 ± 0.8 ‡			40 ± 10 ‡
**DNAIII.FA**	`*CCTCAATTGGTCGTAG`<br>`  GGAGTTAACCAGCATC`		1.6 ± 0.4 ‡			

*DNA used for FA has a 5’ 6-FAM (fluorescein)
†determined via NMR
‡determined via fluorescence anisotropy

Supplementary Table 2. Dissociation constants (K_d) of hBRM/BRG1 BRD and AT-BRD for DNA, determined via NMR and fluorescence anisotropy at 20°C, 50mM potassium phosphate pH 7.0, 50mM KCl, 0.5mM EDTA, 1mM DTT. Shown are the average values over 3-4 replicates and the associated standard deviation.

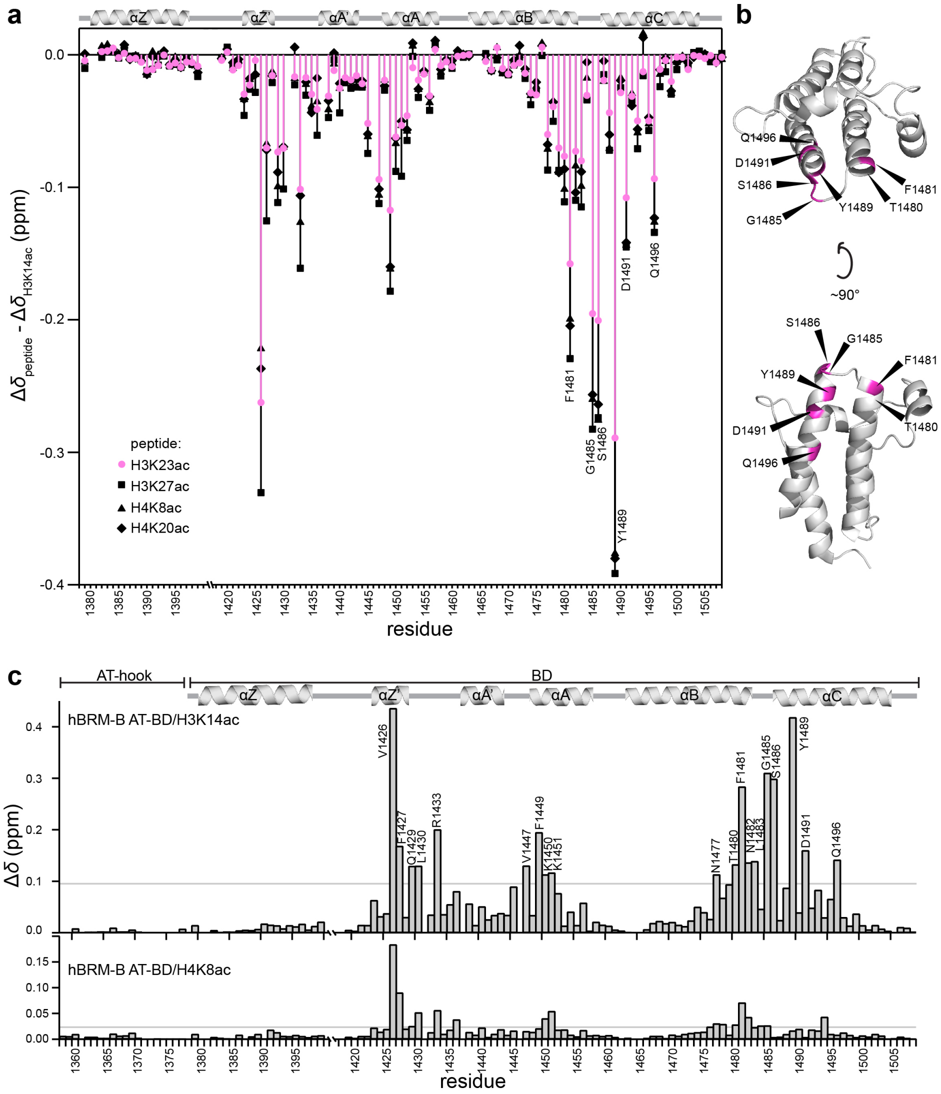

Supplementary Figure 1. Comparison of CSPs for weaker binding acetylated peptides to H3K14ac. a) Plot of $\Delta\Delta\delta$, which corresponds to the difference between the CSPs upon binding H3K14ac to ^{15}N-hBRM BRD and the CSPs induced upon binding the other four acetylated histone tail peptides that were tested to ^{15}N-hBRM-B BRD, as a function of residue. The CSPs for individual histone peptides interacting with ^{15}N-hBRM-B BRD are shown in **Fig. 1c**. A cluster of BRD residues that show a greater similarity between H3K14ac and H3K23ac than the other acetylated histone peptides tested are labeled. **b)** Residues labeled in **(a)** are colored in pink on a structure of hBRM BRD (PDB ID 2DAT) and labeled, along with T1480. **c)** Normalized CSPs for NMR titrations of acetylated peptides into ^{15}N-hBRM-B AT-BRD plotted as a function of residue for H3K14ac and H4K8ac for the protein:peptide molar ratio of 1:19-20. The secondary structure of the BRD is denoted above the plots, and residues that were perturbed greater than the average plus two standard deviations after trimming off the top 10% of CSPs are labeled for binding to H3K14ac. A grey line marks this level of significance for each titration. BRD residues are perturbed the same whether or not the AT-hook is included in the hBRM construct (compare to **Fig. 1c**).

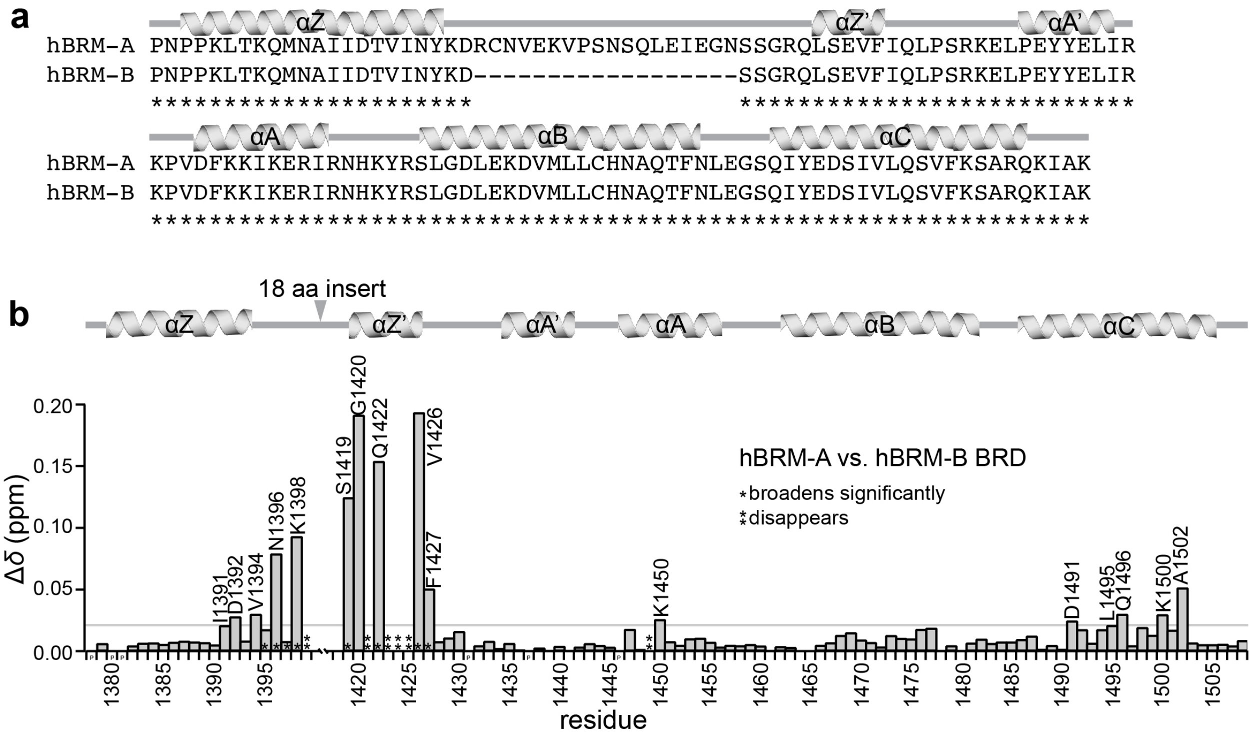

Supplementary Figure 2. The hBRM BRD splice variants show subtle differences in structure and dynamics. a) Sequence alignment (Clustal Omega) of the hBRM-A (residues 1379-1508) and hBRM-B BRD (residues 1379-1399 and 1418-1508) constructs used in this study. Identical (*) residues are marked. The secondary structure of the BRD is labeled above the sequence for reference. **b)** Normalized CSPs of the hBRM-A BRD as compared to the hBRM-B BRD are plotted as a function of residue number, corresponding to the spectra in **Fig. 2a**. The secondary structure of the BRD is labeled above the plots. Residues that are significantly perturbed (greater than the average plus two standard deviations after trimming off the top 10% of CSPs) between the two splice variants are labeled with their residue number. A grey line marks this level of significance. Residues that broaden significantly between splice variants (*) and that broaden beyond detection (**) are labeled.

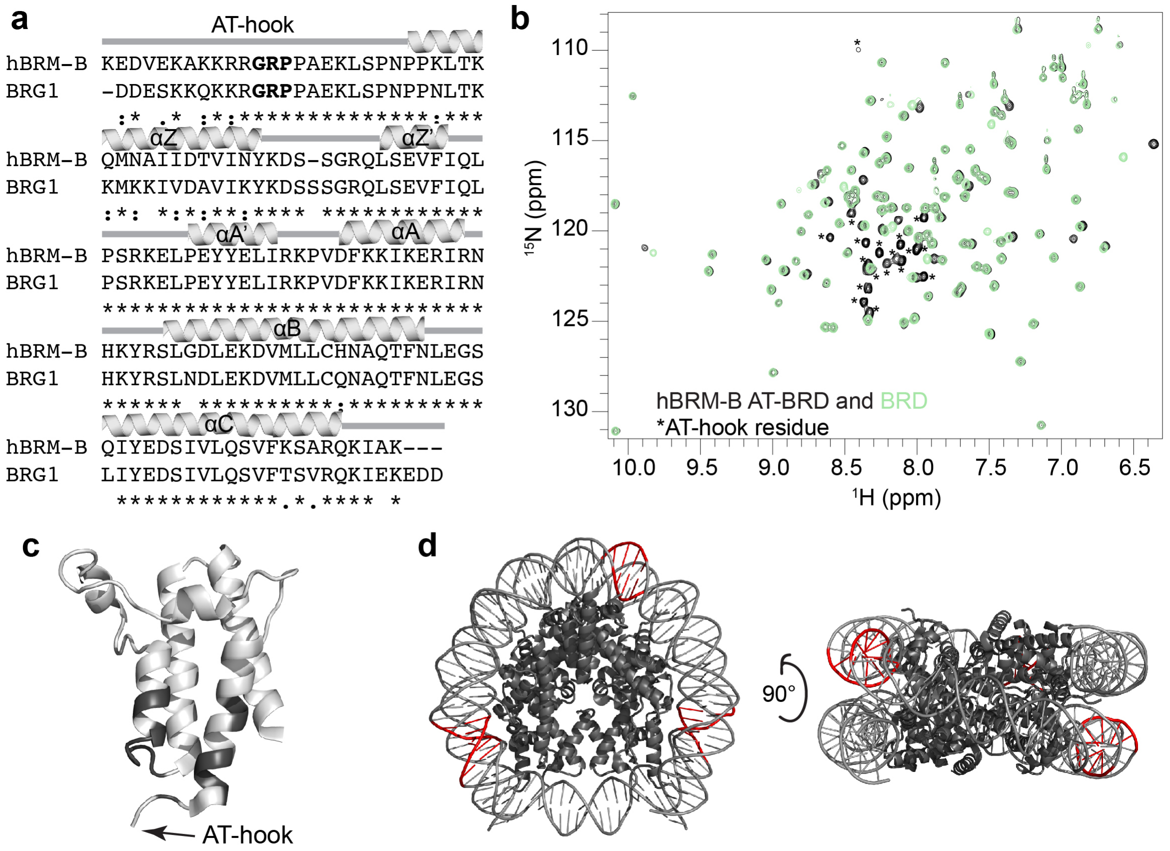

Supplementary Figure 3. The AT-hook does not alter the structure of the BRD. a) Sequence alignment (Clustal Omega) of the BRM-B and BRG1 AT-BRD constructs used in this study. Identical (*) and conserved (:) residues are marked. The secondary structure of the BRD is labeled above the sequence for reference. **b)** Overlay of ^{1}H-^{15}N HSQC spectra of the BRM-B BRD (green) and AT-BRD (black). Resonances corresponding to AT-hook residues are labeled (*). **c)** Cartoon representation of the hBRM BRD structure (PDB ID 2DAT) with residues that are significantly perturbed in the presence of the AT-hook colored in dark grey. The N-terminus where the AT-hook attaches is pointed out by the arrow **d)** Cartoon representation of the structure of a nucleosome formed with Widom 601 DNA (PDB ID 3LZ0) with canonical AT-rich binding sites of AT-hooks highlighted in red.

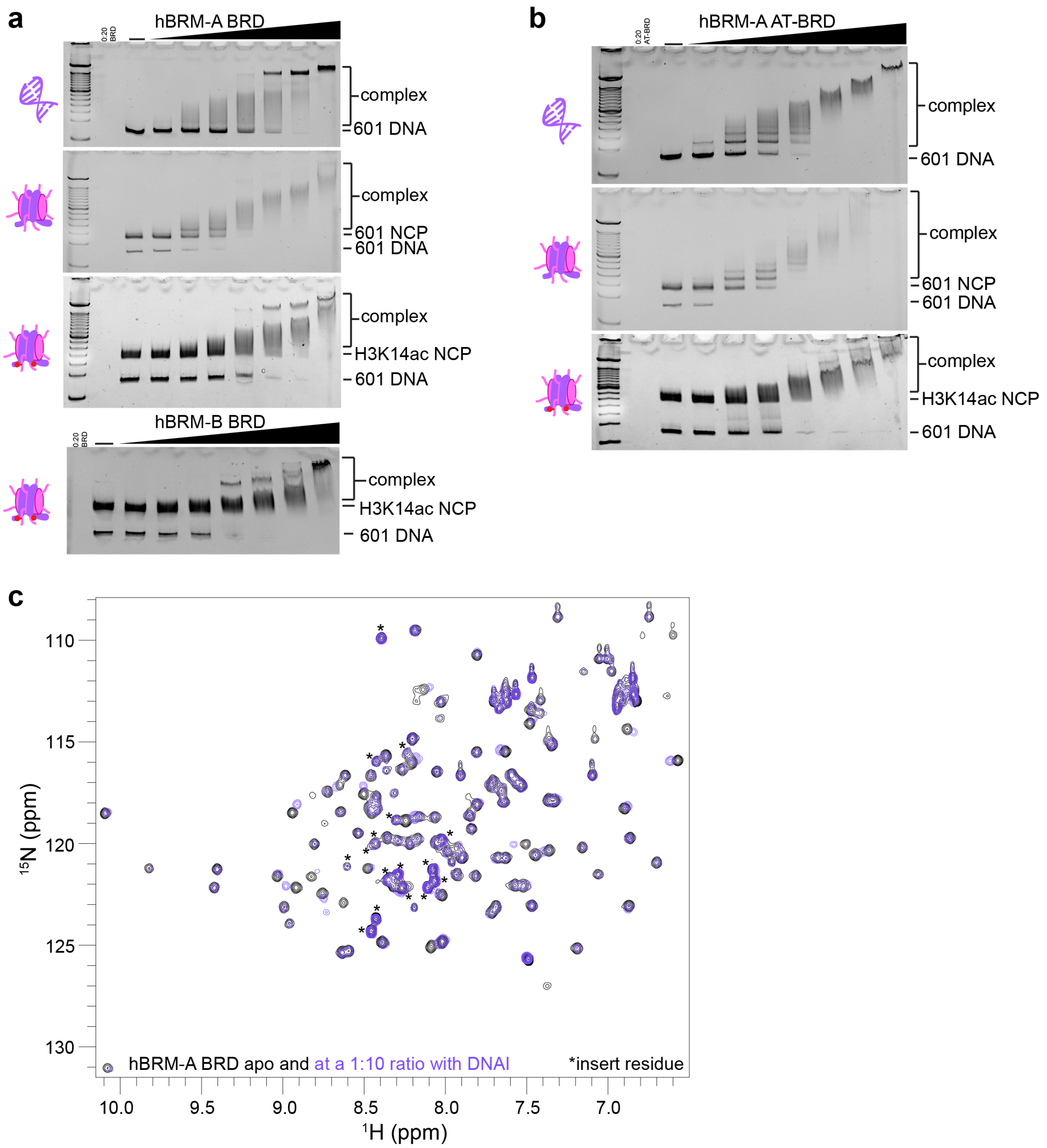

Supplementary Figure 4. EMSAs with the hBRM-A splice variant binding to 601 DNA and 601 NCP. a) EMSAs carried out with the hBRM-A BRD splice variant and Widom 601 DNA, 601 NCP, or H3K14ac-modified 601 NCP. See **Fig. 3a** and the bottom gel here for comparison with the hBRM-B BRD. Gels were run with a 100bp DNA ladder on the left and stained with ethidium bromide for visualization. **b)** EMSAs carried out with the hBRM-A AT-BRD splice variant and Widom 601 DNA, 601 NCP, or H3K14ac-modified 601 NCP. See **Figs. 4a** and **6a** for comparison with the hBRM-B AT-BRD. Gels were run with a 100bp DNA ladder in the far left lane and were stained with ethidium bromide for visualization. **c)** Overlay of ^{1}H-^{15}N HSQC spectra of ^{15}N-hBRM-A BRD apo (black) and at a 1:10 ratio with DNAI (purple). Resonances corresponding to the extra 18 aa in hBRM-A are labeled (*). It is clear that the 18-aa insert does not interact with DNA.

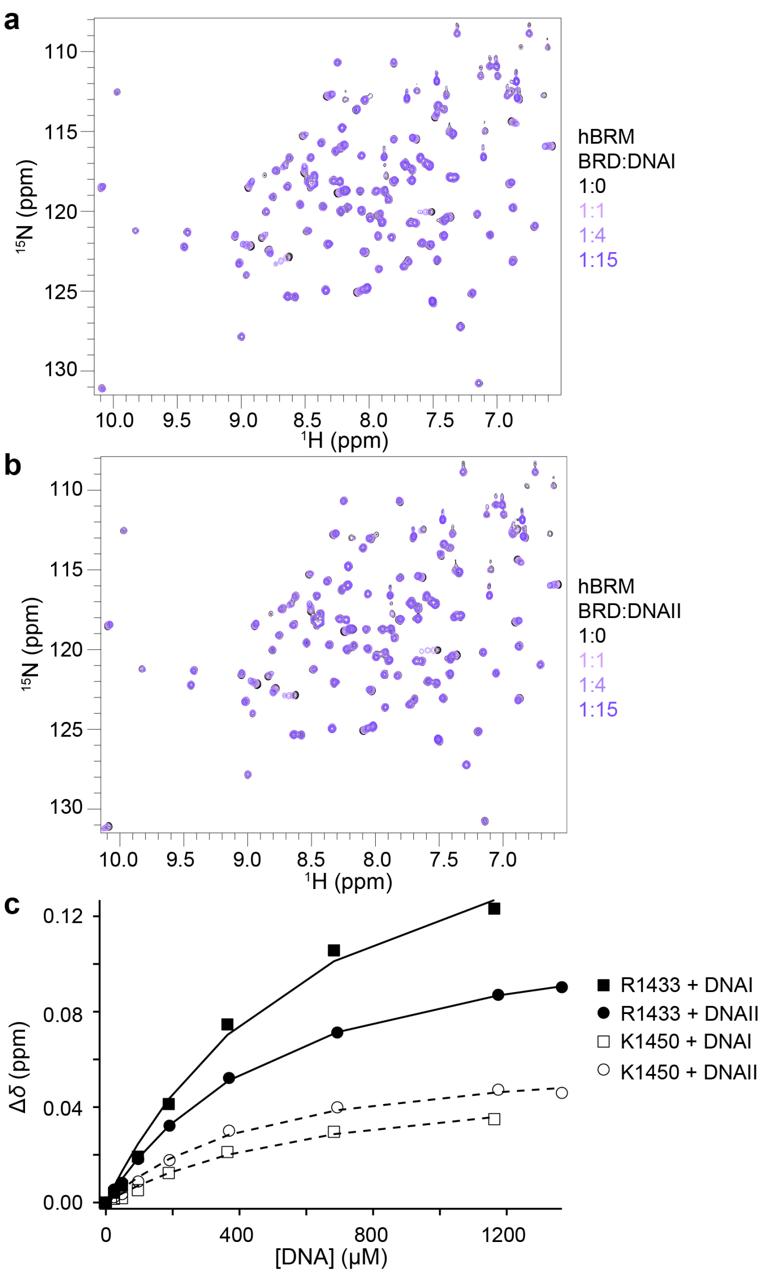

Supplementary Figure 5. Titration of DNA into the hBRM-B BRD. Overlay of ^{1}H-^{15}N HSQC spectra of ^{15}N-hBRM BRD upon titration of DNAI **(a)** or DNAII **(b)**. These are the full spectra corresponding to **Fig. 3b**, and spectra are color coded according to protein:DNA molar ratio as shown in the legend. DNA titrations were collected at protein:DNA molar ratios of 1:0, 1:0.25, 1:0.5, 1:1, 1:2, 1:4, 1:8, and 1:15 (additionally 1:18 for DNAII). For clarity, only 4 points are displayed. The global decrease in intensity/line broadening with increasing concentration of DNA is likely due to an increase in rotational correlation time as the DNA size is significantly larger as compared to that of the BRD. **c)** Representative binding curves for the titration of DNA into ^{15}N-hBRM-B BRD, corresponding to **Fig. 3b,c**. Binding curves are shown for R1433 (closed symbols) and K1450 (open symbols) as a function of DNAI (squares) or DNAII (circles) concentration.

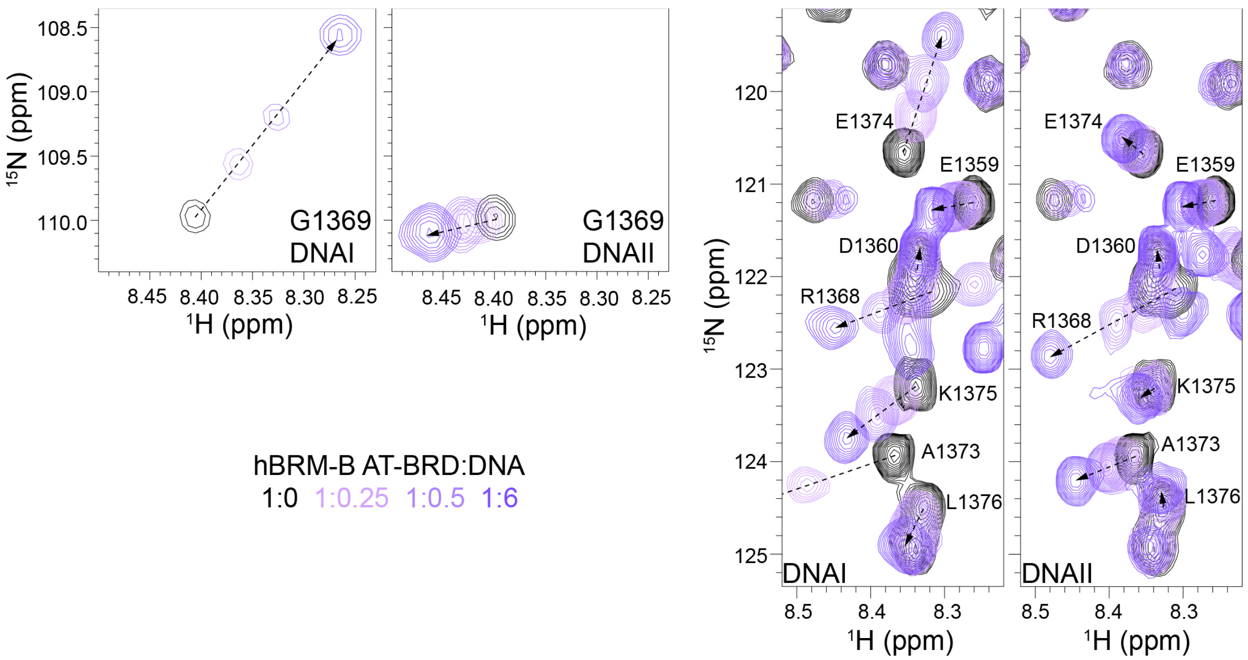

Supplementary Figure 6. Distinct bounds states of the AT-hook. Overlay of ^{1}H-^{15}N HSQC spectra of wild type ^{15}N-hBRM-B AT-BRD upon titration of DNAI or DNAII, as labeled. Spectra are color coded according to protein:DNA molar ratio as shown in the legend. Titrations were collected at protein:DNA molar ratios of 1:0, 1:0.25, 1:0.5, 1:1, 1:1.5, 1:3, and 1:6. For clarity, only 4 points are displayed. The trajectories of resonances corresponding to AT-hook residues are followed with dashed arrows. Note that G1369 (of the GRP), E1374, and L1376 have distinct chemical shift values in DNAI-bound as compared to DNAII-bound states, suggestive of a distinct bound state.

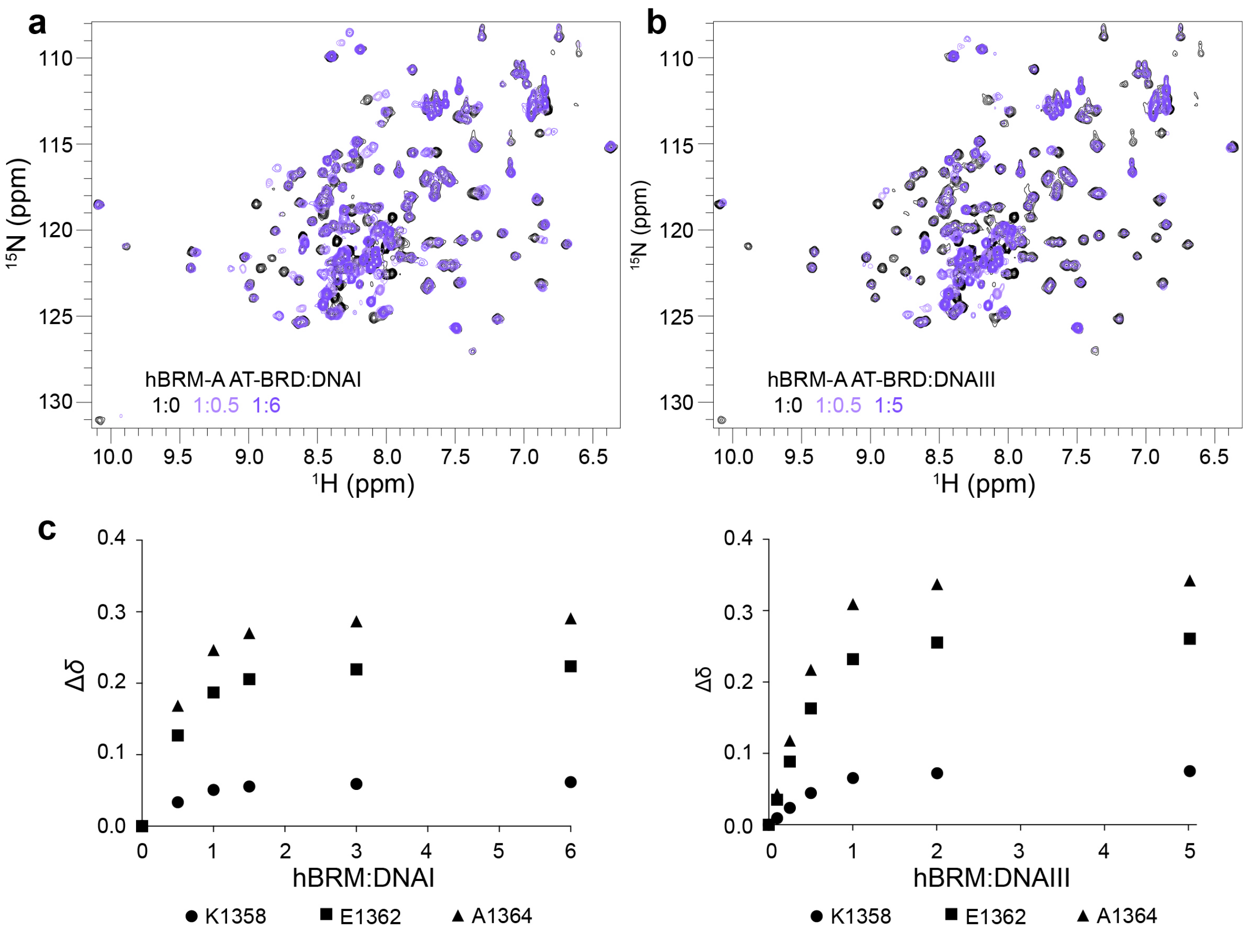

Supplementary Figure 7. Comparison of titrations with 10 and 15bp DNA into the hBRM-A AT-BRD. Overlay of ^{1}H-^{15}N HSQC spectra of ^{15}N-hBRM-A AT-BRD upon titration of DNAI **(a)** or DNAIII **(b)**. Spectra are color coded according to protein:DNA molar ratio as shown in the legend. DNAI titrations were collected at protein:DNA molar ratios of 1:0, 1:0.5, 1:1, 1:1.5, 1:3, and 1:6, and DNAIII titrations were collected at ratios of 1:0, 1:0.1, 1:0.25, 1:0.5, 1:1, 1:2, and 1:5. Protein concentration was 100 μM. For clarity, only 3 points are displayed. **c)** Representative binding curves for the titration of DNAI (left) DNAIII (right) into ^{15}N-hBRM-A AT-BRD for residues in the AT-hook (left) and residues in the BRD (right). This data was collected under stoichiometric conditions and indicates that the AT-BRD binds both DNAI and DNAIII at a 1:1 stoichiometry under these conditions.

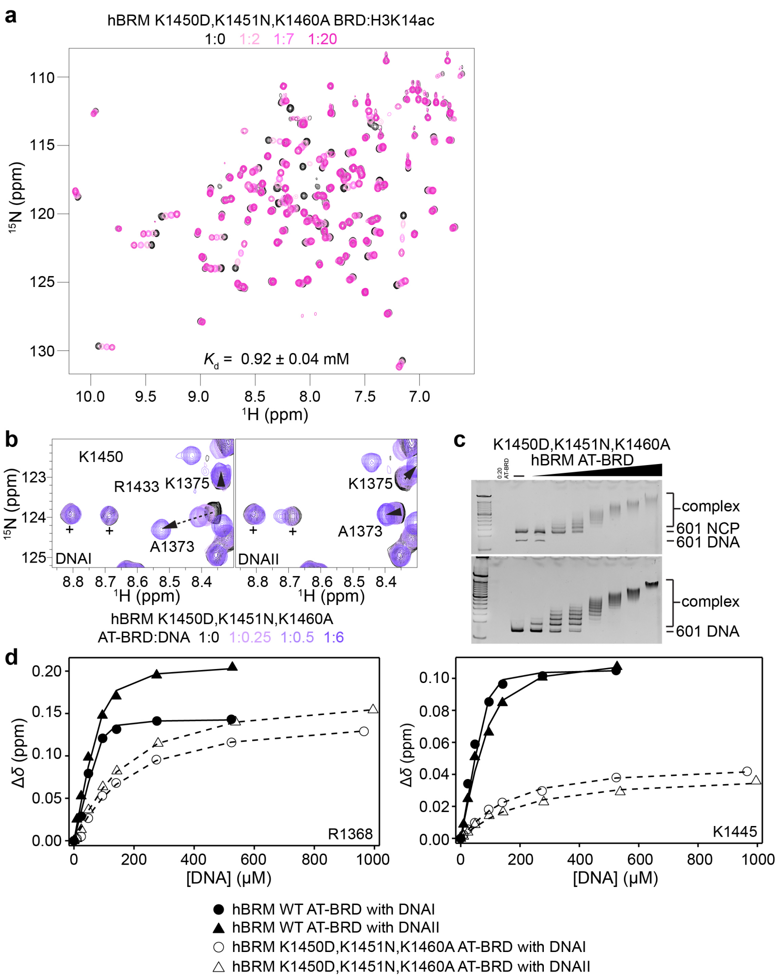

Supplementary Figure 8. **AT-BRD binds DNA significantly weaker when the basic patch of the BRD is mutated. a)** Overlay of ^{1}H-^{15}N HSQC spectra for ^{15}N-hBRM K1450D,K1451N,K1460A BRD upon titration of H3K14ac. The spectra are color coded for protein:H3K14ac molar ratio as shown in the legend. H3K14ac titrations were collected at protein:H3K14ac molar ratios of 1:0, 1:0.5, 1:1, 1:2, 1:4, 1:7, 1:12 and 1:20. For clarity, only 4 points are displayed. The dissociation constant displayed is the average and standard deviation from 16 significantly perturbed residues. **b)** Overlay of ^{1}H-^{15}N HSQC spectra for ^{15}N-hBRM K1450D,K1451N,K1460A AT-BRD upon titration of DNAI (left) or DNAII (right), for comparison with **Fig. 4b**. The spectra are color coded for protein:DNA molar ratio as shown in the legend. DNA titrations were collected at protein:DNA molar ratios of 1:0, 1:0.1, 1:0.25, 1:0.5, 1:1, 1:1.5, 1:3, 1:6 and 1:12. For clarity, only 4 points are displayed. The (+) marking indicates peaks corresponding to the mutated residues. **c)** EMSAs carried out with K1450D,K1451N,K1460A AT-BRD and either 601 NCP (top) or free 601 DNA (bottom). Gels were run with a 100bp DNA ladder on the left and stained with ethidium bromide for visualization. **d)** Representative binding curves for the titration of DNA into ^{15}N-hBRM-B WT (solid symbols/lines) and K1450D,K1451N,K1460A (open symbols/lines) AT-BRD. Binding curves are shown for R1368 (left) and K1445 (right) as a function of DNAI (circles) or DNAII (triangles) concentration. This mutant binds DNA two orders of magnitude weaker than WT AT-BRD (see Supplementary Table 2).

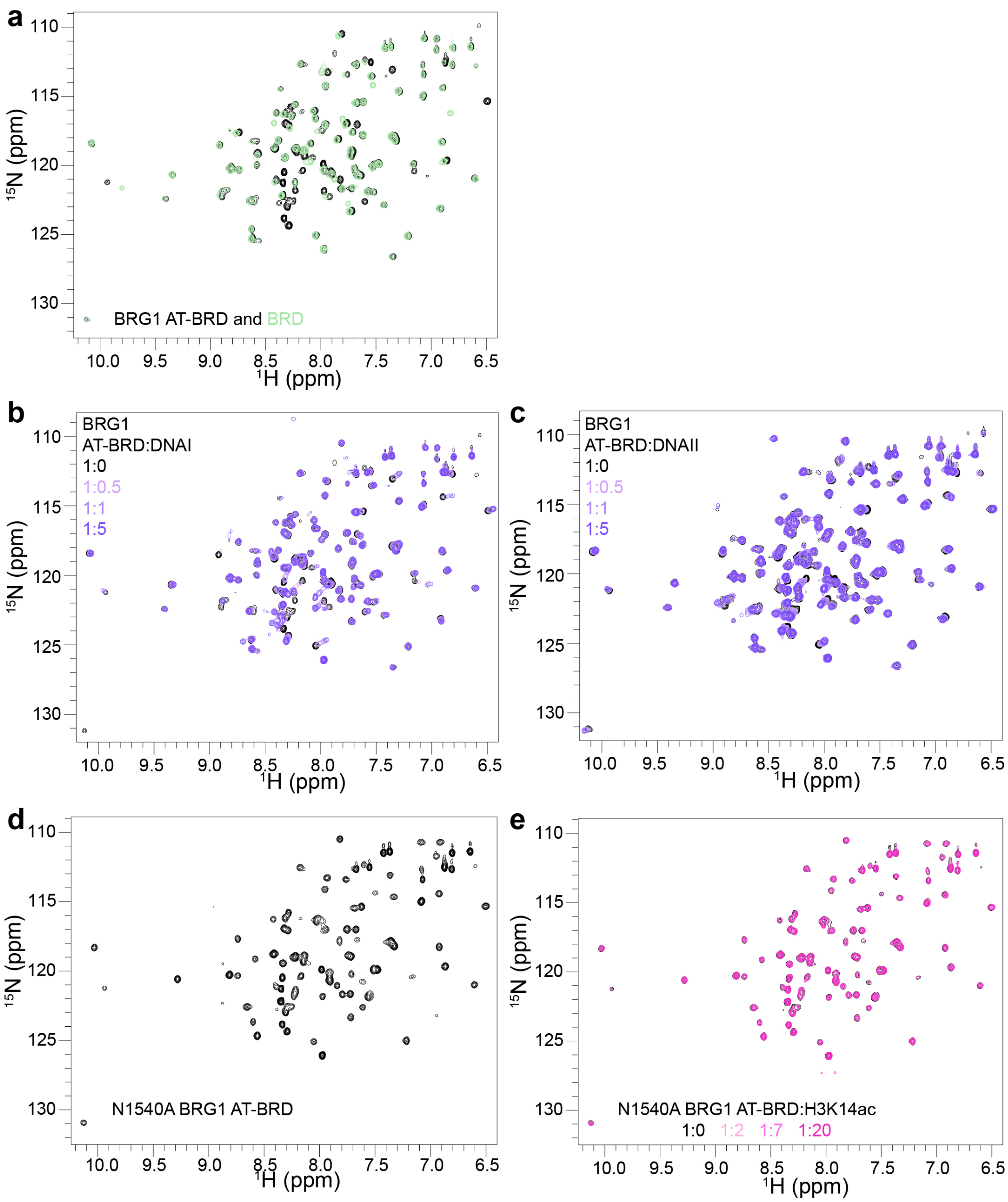

Supplementary Figure 9. Full spectra of ^{15}N-BRG1 AT-BRD. a) ^{1}H-^{15}N HSQC spectrum of ^{15}N-BRG1 AT-BRD (black) and BRD (green). Overlay of ^{1}H-^{15}N HSQC spectra of ^{15}N-BRG1 AT-BRD upon titration of DNAI **(b)** or DNAII **(c)**. These are the full spectra corresponding to **Fig. 4b**, and spectra are color coded according to protein:DNA molar ratio as shown in the legend. DNA titrations were collected at protein:DNA molar ratios of 1:0, 1:0.5, 1:1, 1:2, 1:3, and 1:5 (additionally 1:6 for DNAII). For clarity, only 4 points are displayed. **d)** ^{1}H-^{15}N HSQC spectrum of ^{15}N-N1540A BRG1 AT-BRD, showing that the N1540A mutation does not alter the BRD fold. **e)** Overlay of ^{1}H-^{15}N HSQC spectra of ^{15}N-N1540A BRG1 AT-BRD collected with increasing concentrations of H3K14ac, with the ratio of protein:peptide color-coded as labeled in the legend. These spectra indicate that the N1540A mutation abrogates binding to H3K14ac. All spectra were collected at 25°C on an 800MHz spectrometer.

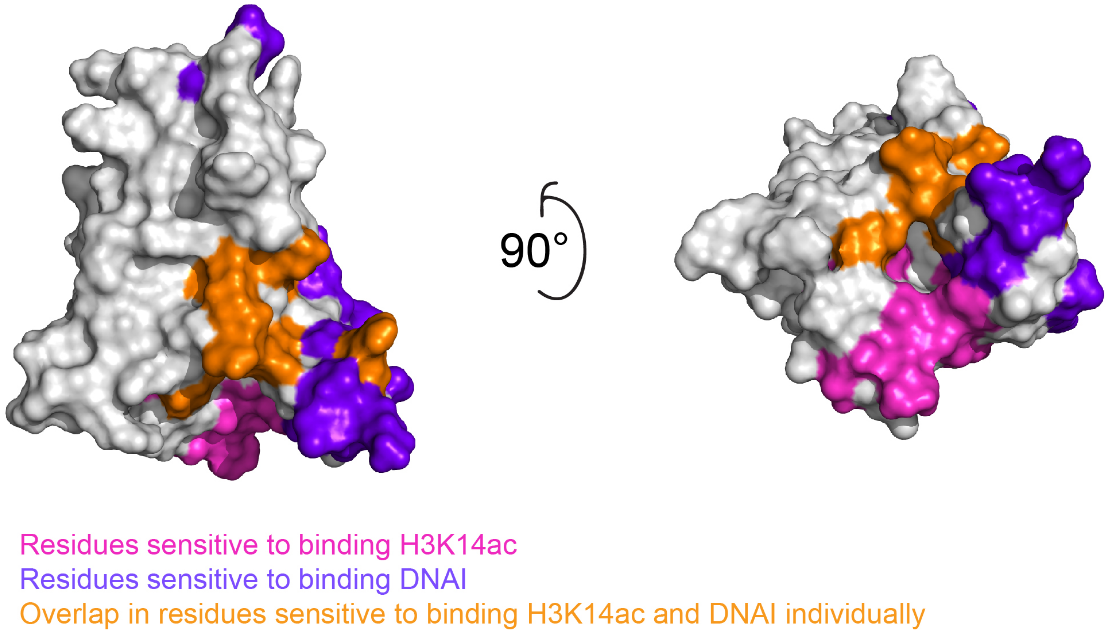

Supplementary Figure 10. Overlap in the residues that are perturbed upon binding H3K14ac and DNA. A surface representation of the hBRM BRD structure (PDB ID 2DAT) with residues colored according to their involvement in binding to H3K14ac peptide (pink), DNA (purple) or both (orange). Important residues were determined from individual titrations of H3K14ac (see **Fig. 1c**) and DNAI (see **Fig. 3d**) into ^{15}N-hBRM-B BRD.

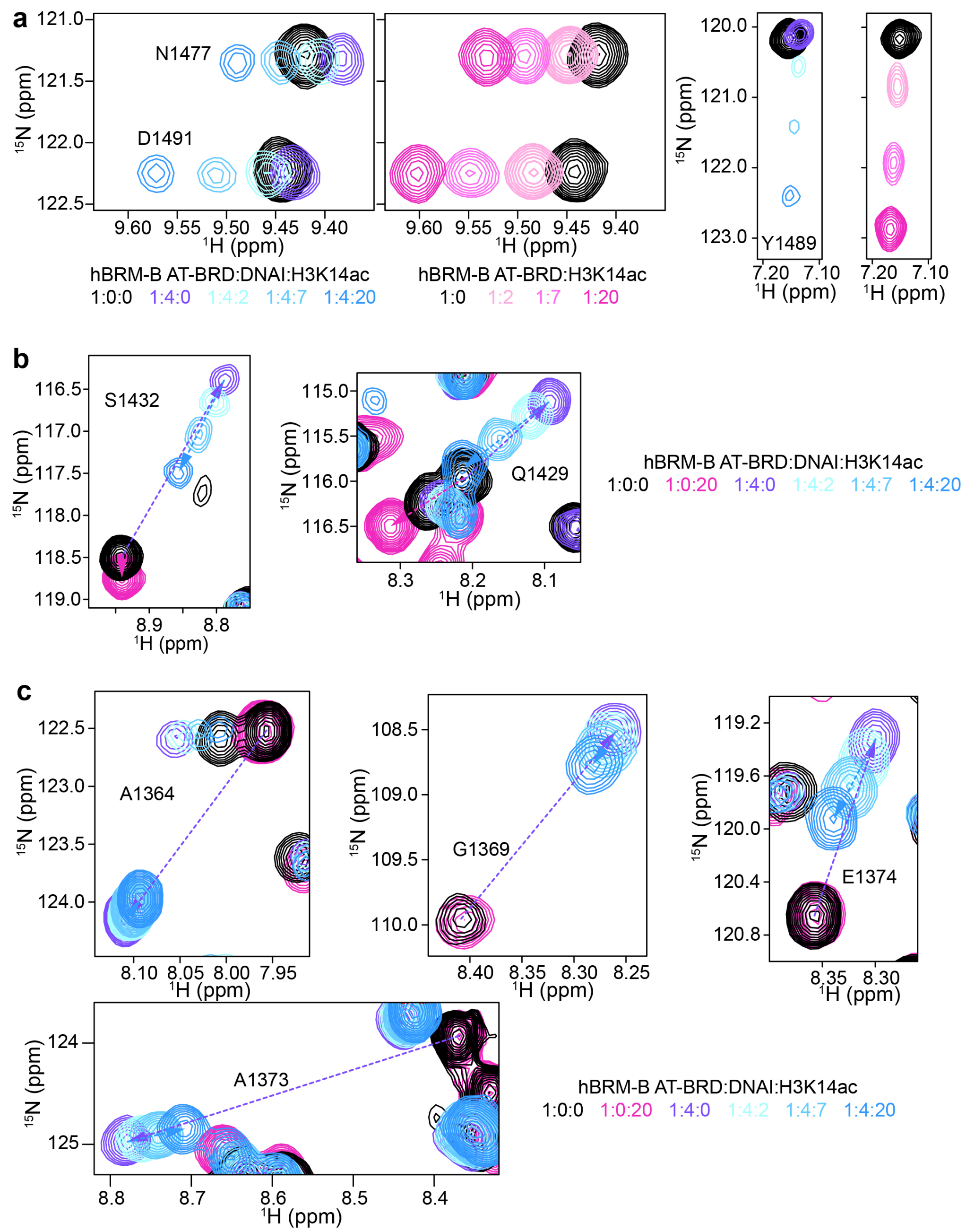

Supplementary Figure 11. The hBRM BRD can bind DNA and H3K14ac simultaneously. Overlay of ^{1}H-^{15}N HSQC spectra of the ^{15}N-hBRM-B AT-BRD apo (black) in the presence of H3K14ac (pink), DNAI (purple), or both DNA and peptide (blue). For clarification, in some panels dashed arrows trace the linear trajectory between apo and DNAI-bound (purple) and DNAI-bound and both DNAI- and H3K14ac-bound AT-BRD (blue). **a**) Resonances for residues N1477, Y1489, and D1491 are not significantly perturbed by DNAI-binding, but sense the binding of H3K14ac independent of whether DNAI is pre-bound. **b**) S1432 and Q1429 move toward the H3K14ac-bound state when H3K14ac is titrated into DNAI-bound AT-BRD. **c**) The AT-hook (see A1364 and G1369) remains bound to DNAI upon titration of H3K14ac, and residues A1373 and E1374 are sensitive to formation of the ternary complex.

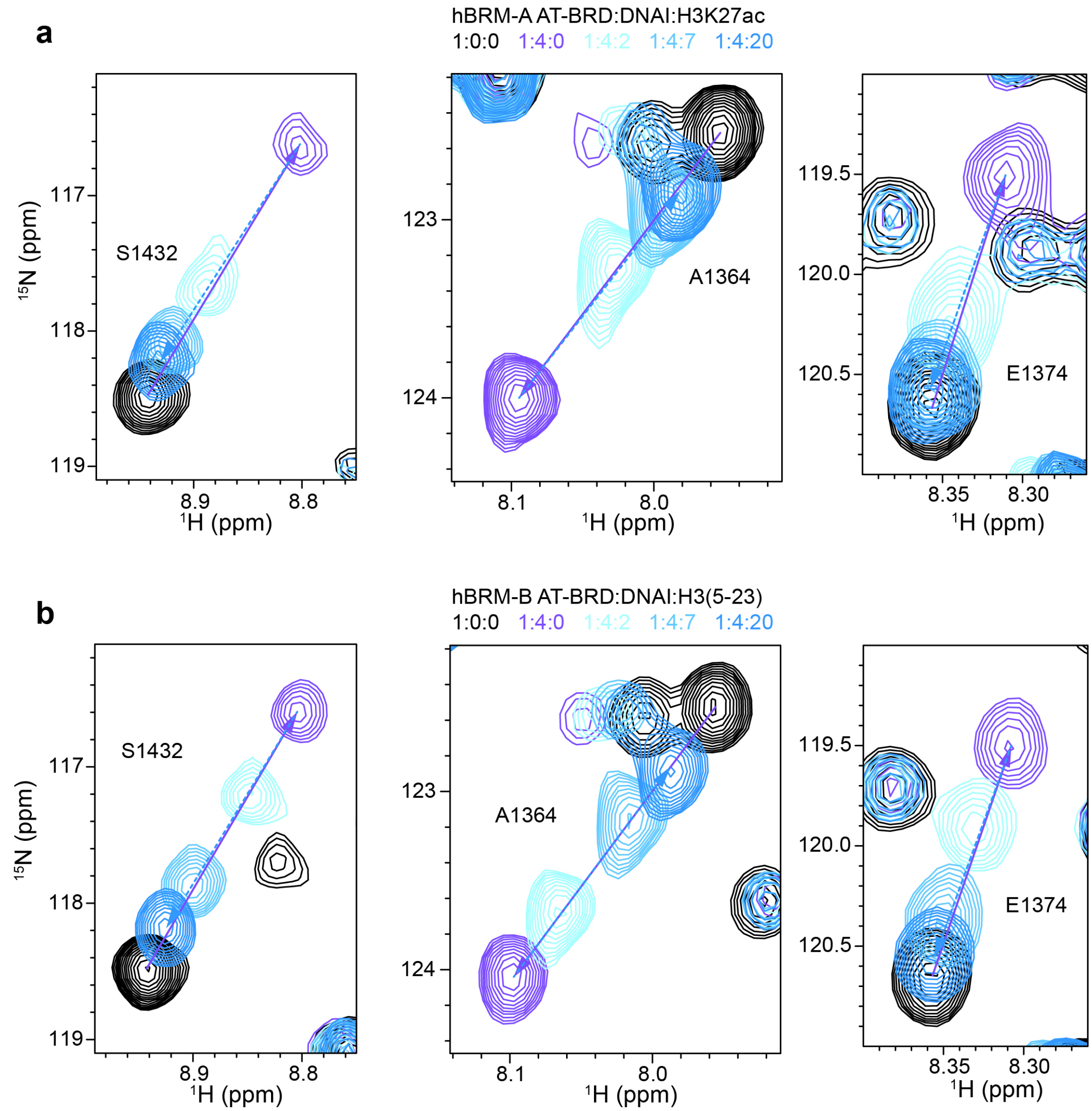

Supplementary Figure 12. H3K27ac and unmodified H3 compete DNA off of hBRM BRD through direct peptide-DNA interaction. a) Overlay of ^{1}H-^{15}N HSQC spectra of the ^{15}N-hBRM-A AT-BRD apo (black) in the presence of DNAI (purple) or both DNA and H3K27ac peptide (shades of blue according to legend). **b)** Overlay of ^{1}H-^{15}N HSQC spectra of the ^{15}N-hBRM-B AT-BRD apo (black) in the presence of DNAI (purple) or both DNA and unmodified H3(5-23) peptide (shades of blue according to legend). Addition of either peptide to DNAI-bound AT-BRD causes the AT-hook and BRD to release the DNAI, as shown by the movement of resonances back toward the apo state. For clarification, arrows trace the linear trajectory between apo and DNAI-bound AT-BRD (purple) and upon titration of peptide into DNAI-bound AT-BRD (blue).

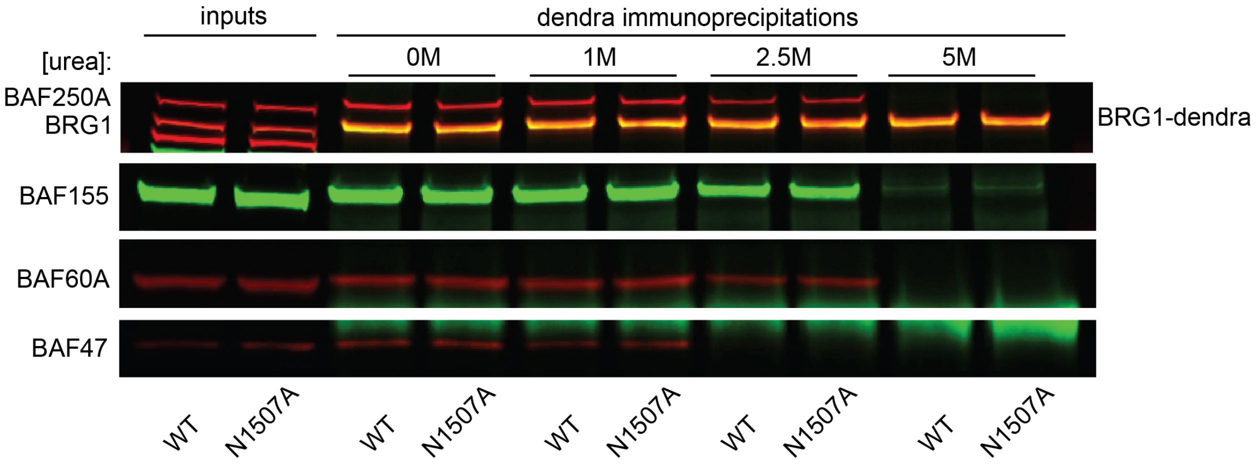

Supplementary Figure 13. BRD mutation does not alter the BAF complex stability. Western blot of BAF complex subunits following immunoprecipitation of Dendra2-tagged wild type BRG1 or BRG1(N1507A) protein from urea-denatured nuclear lysates. Increasing urea concentrations and ESC line indicated.

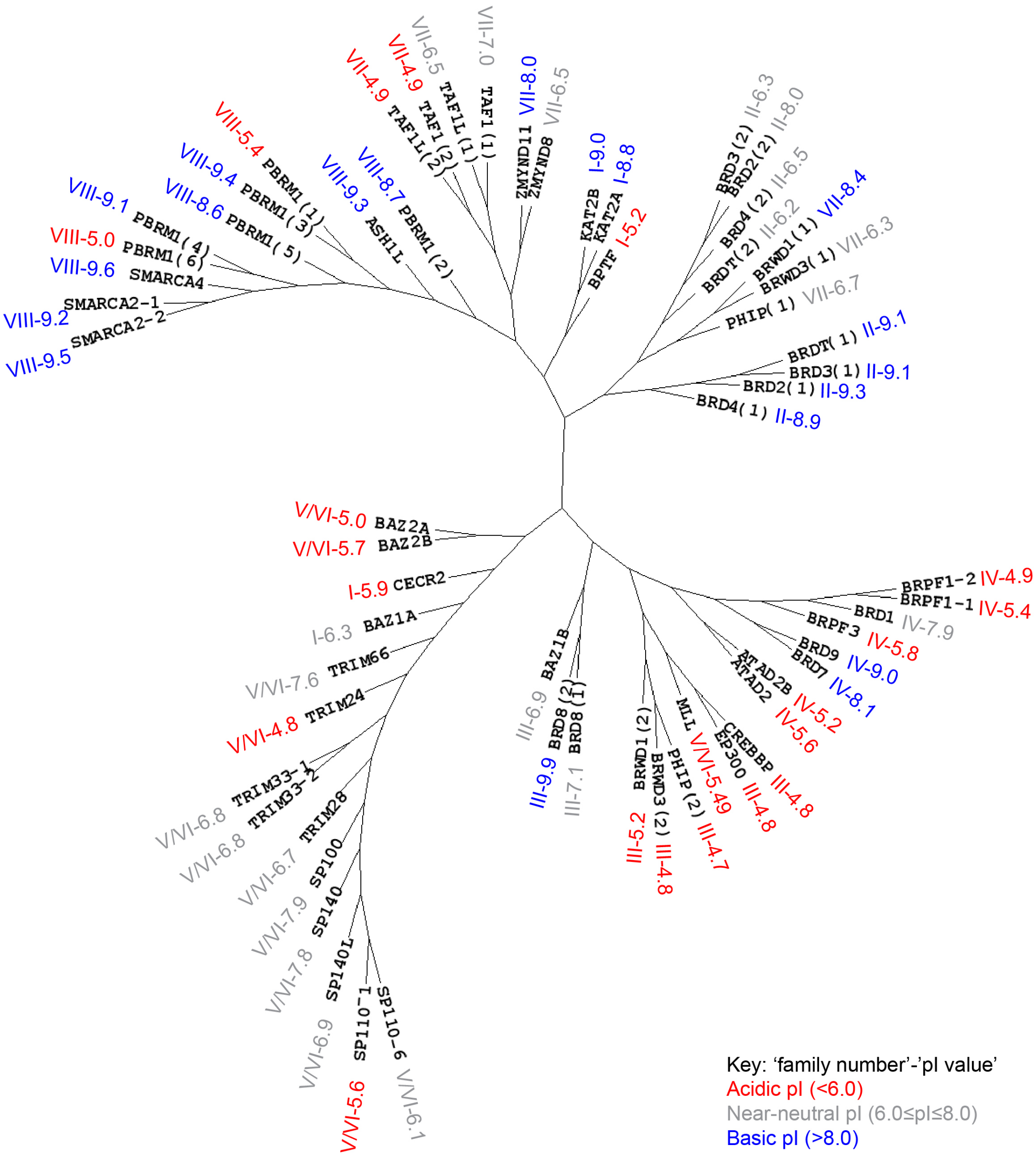

Supplementary Figure 14. A third of identified BRDs are likely to associate with DNA. A phylogenetic tree of all human bromodomains created using ChromoHub. Proteins with multiple BRDs have the BRD number labeled in parentheses. Proteins with insertions and deletions occurring within the BRD have the splice variant number listed following a hyphen. The protein name is then labeled with the family number (in Roman numerals) followed by the theoretical pI value (calculated using ExPASY). These are color-coded for acidic pI values (pI<6.0, red), near-neutral pI values (6.0≤pI≤8.0), and basic pI values (pI>8.0, blue).

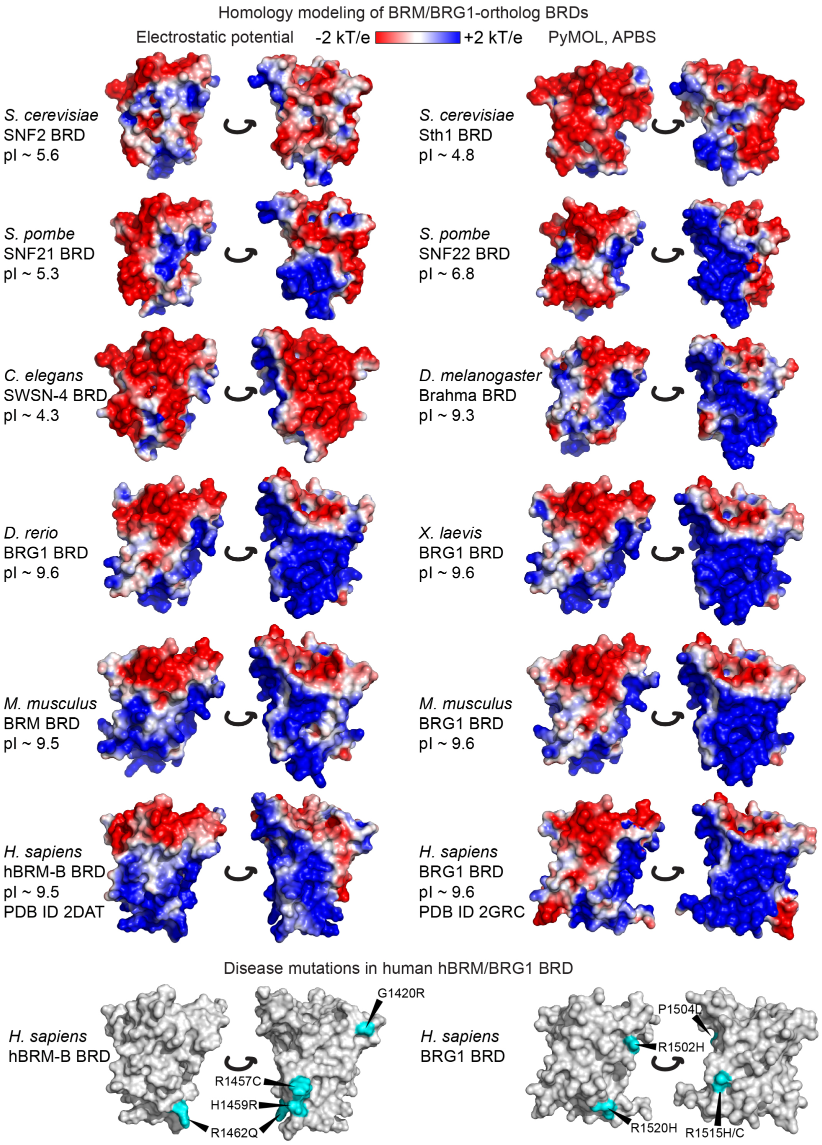

Supplementary Figure 15. Evolution of the basic patch on hBRM/BRG1 BRD and cancer mutations. (Top) The surface electrostatics are shown for modeled BRDs of SNF2-like ATPase subunits of the SWI/SNF-like complexes for commonly-studied model organisms representing different evolutionary stages. The BRDs for the non-human species were modeled onto a structure of the BRG1 BRD (PDB ID 2GRC) using SWISS-MODEL. Electrostatic surfaces were depicted in PyMOL using APBS. Theoretical pI values (calculated using ExPASY) are shown for all BRDs. A high pI value and basic surface patches suggest the possibility of DNA-binding activity. Analysis of the predicted surface electrostatics of the BRDs for orthologs of hBRM/BRG1 reveal that the DNA-binding property of the BRD is an evolved function, and the first appearance of a basic patch among the analyzed organisms is on the BRD in drosophila BRM. (Bottom) Several cancer mutations (highlighted in cyan, identified using cBioPortal) have been identified in the basic patch on BRG1 and hBRM, as well as one mutation associated with Nicolaides-Baraitser syndrome that is just adjacent to this patch, suggesting that this region contributes to BRG1/hBRM function and possibly tumor suppression.

BRM BRD with 601 DNA

a

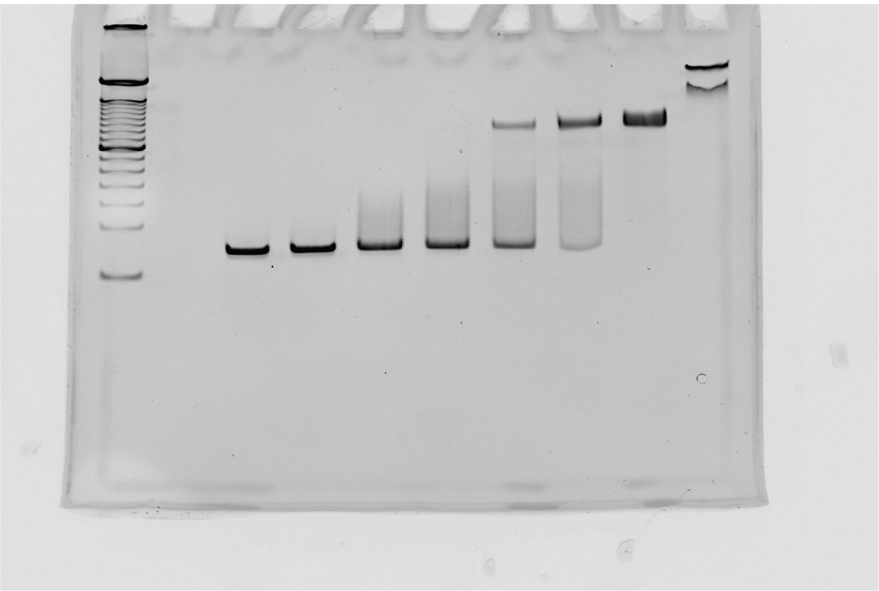

BRM BRD with 601-NCP

b

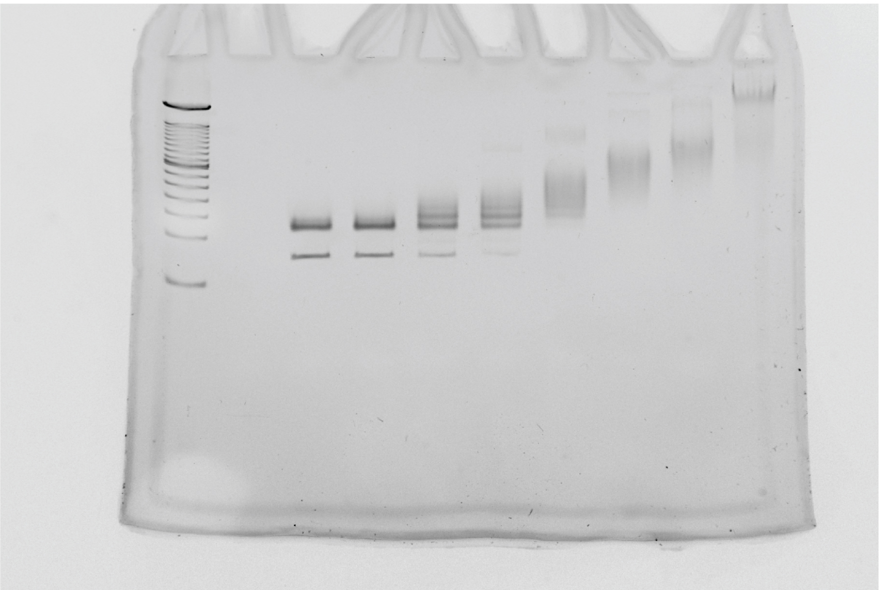

Supplementary Figure 16. Representative uncropped EMSA gels. Representative uncropped gels for EMSAs carried out with hBRM-BRD and either 601 DNA **(a)** or free 601 NCP **(b)**. Gels were run with a 100bp DNA ladder on the left and stained with ethidium bromide for visualization. Compare to cropped EMSAs in **Fig. 3a**.
